# P-2128. First-in-human ^18^F-Fluorodeoxysorbitol PET for Noninvasive Diagnosis of Invasive Fungal Infections

**DOI:** 10.1093/ofid/ofae631.2284

**Published:** 2025-01-29

**Authors:** Carlos E Ruiz-Gonzalez, Oscar J Nino-Meza, Medha Singh, Yuderleys Masias-Leon, Amy Kronenberg, Xueyi Chen, Elizabeth Tucker, Olivia S Kates, Sean Zhang, Sanjay K Jain

**Affiliations:** Johns Hopkins University/School of Medicine, Glen Burnie, Maryland; Johns Hopkins University School of Medicine, Baltimore, Maryland; Johns Hopkins University, Baltimore, Maryland; Johns Hopkins University School of Medicine, Baltimore, Maryland, United States., Baltimore, MD; Johns Hopkins, Baltimore, Maryland; Johns Hopkins University School of Medicine, Baltimore, Maryland; Johns Hopkins, Baltimore, Maryland; Johns Hopkins University, Baltimore, Maryland; Johns Hopkins Hospital, Baltimore, Maryland; Johns Hopkins Children's Center, Baltimore, MD

## Abstract

**Background:**

Invasive mold infections are increasingly prevalent in immunosuppressed patients and associated with high mortality. Definitive diagnosis is challenging and may require invasive procedures (bronchoalveolar lavage, biopsy) or rely on biomarkers, e.g., galactomannan, which has limited sensitivity for *Aspergillus* and does not detect other mold species. There is need for sensitive, noninvasive tools to diagnose invasive mold infections. Molds, but not mammalian cells, metabolize sorbitol; recent studies suggest that ^18^F-fluorodeoxysorbitol (^18^F-FDS), a fluoro-analog of sorbitol, could be developed as a diagnostic tool for invasive mold infections.

**Figure 1.**
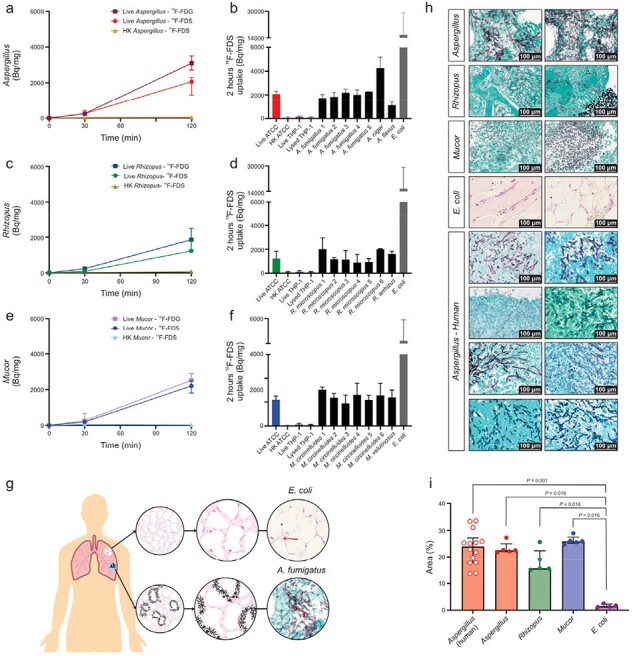
18F-FDS uptake in molds.

18F-FDS and 18F-FDG uptake in Aspergillus (panels a-b), Rhizopus (panels c-d), and Mucor (panels e-f) performed at least in triplicate, demonstrating substantial and rapid uptake by the laboratory as well as all clinical isolates (120 min), suggesting that this is a conserved process across molds. No uptake is noted in heat killed organisms or mammalian cells, demonstrating that the uptake is not due to nonspecific binding. (g) Schematic representation of the tissue mass occupied by a bacterial versus fungal infection. Representative tissue histology from mice (panel h) and human tissues (eight unique patients with pulmonary, cerebral or rhinosinusal invasive aspergillosis) demonstrating that fungi constitute a significantly higher mass (15.6%-25.9%) of the infected lesions versus E. coli infections (1.5%) (panel i) and therefore can be visualized well in vivo even though they have lower uptake than E. coli. Data are expressed as median and interquartile range (IQR). Statistical analyses were performed using a two-tailed Mann-Whitney U test. Histology staining: GMS-Grocott Methenamine Silver for fungi and Gram stain for bacteria. Abbreviations: Bq/mg= Becquerel per milligram of protein; HK: Heat-killed; ATCC: American Type Culture Collection.

**Methods:**

We tested ^18^F-FDS uptake by clinical isolates of relevant mold pathogens; evaluated ^18^F-FDS positron emission tomography (PET) to detect invasive infections with *Aspergillus*, *Rhizopus,* and *Mucor* in clinically relevant mouse models; and performed first-in-human ^18^F-FDS PET in patients with invasive aspergillosis (NCT05611892).

**Figure 2.**
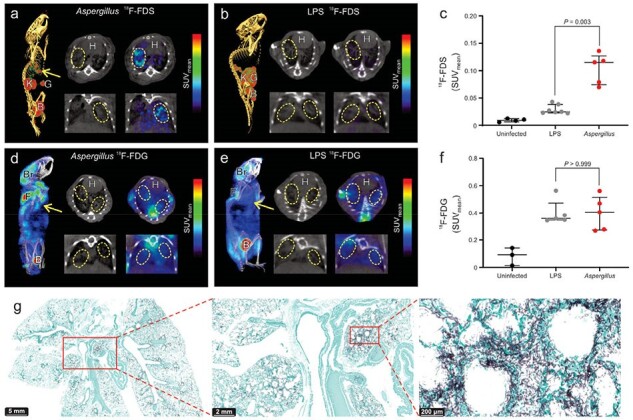
PET/CT in mouse models of pulmonary aspergillosis.

18F-FDS PET/CT can detect and localize infectious lesions due to pulmonary aspergillosis in immunosuppressed mice which colocalize well with the lesions noted on CT (panel a). However, even though lung disease is noted on the CT in LPS-induced sterile inflammation in mice, no 18F-FDS PET signal is noted (panel b). (c) 18F-FDS PET signal quantified as SUVmean shows significantly higher activity at fungal infection sites (n = 5 mice) versus sterile inflammation (n = 7 mice) (P = 0.003). Similar experiments were performed with 18F-fluorodeoxyglucose (18F-FDG) (n = 5 mice per group). As shown in panels d-f, 18F-FDG PET cannot distinguish fungal lesions (P > 0.999) from sterile inflammation as 18F-FDG is a nonspecific marker of inflammation. (g) GMS staining in lung samples from mice infected with Aspergillus at different magnification levels (Sidebars: 5 mm, 2 mm, and 200µm). Lesions are highlighted with arrows and outlined in yellow. Data are expressed as median and interquartile range (IQR). Statistical analyses were performed using a two-tailed Mann-Whitney U test. Abbreviations: SUVmean: Standardized uptake value mean. LPS: Lipopolysaccharide. B: bladder. Br: brain. F: fat tissue. G: gastrointestinal tract. H: heart. K: kidneys.

**Results:**

^18^F-FDS was rapidly accumulated by laboratory and all clinical strains of molds tested, with no uptake in heat-killed fungi or mammalian cells (Fig. 1). ^18^F-FDS PET was able to detect and localize infectious lesions due to pulmonary aspergillosis in immunosuppressed mice and differentiate them from LPS-induced sterile inflammation (**Fig. 2**); and to detect and localize rhinosinusal infections due to *Aspergillus*, *Rhizopus,* and *Mucor*; as well as cerebral aspergillosis (**Fig. 3**). Finally, first-in-human ^18^F-FDS PET in three patients with invasive pulmonary or cerebral aspergillosis was safe and detected infectious lesions (target-to-nontarget ratio > 3), including in a patient with an azole-resistant infection (**Fig. 4**).

**Figure 3.**
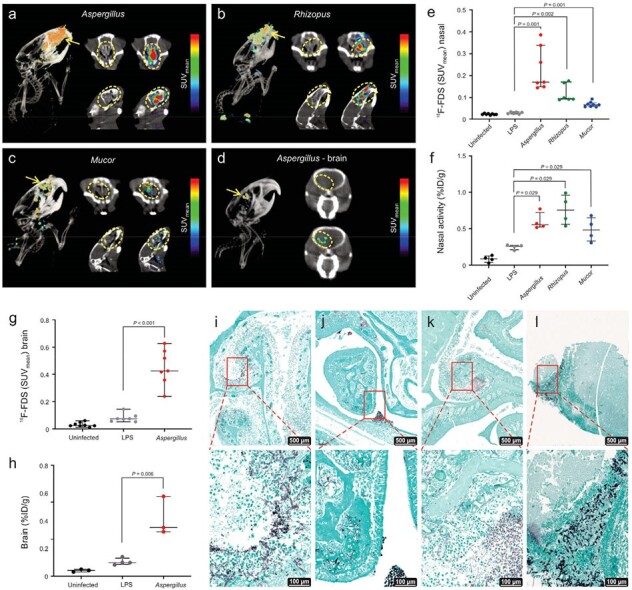
Extrapulmonary mouse models of invasive fungal infections.

18F-FDS PET/CT can detect and localize invasive rhinosinusal infections in immunosuppressed mice due to Aspergillus (panel a), Rhizopus (panel b) and Mucor (panel c) and cerebral aspergillosis (panel d). 18F-FDS PET signal quantified as SUVmean shows significantly higher activity at fungal rhinosinusal and brain infection sites respectively (panels e and g) versus sterile inflammation, which is confirmed using postmortem ex vivo gamma counting (panels f and h). GMS staining for fungi in mice with rhinosinusal infection by Aspergillus (panel i), Rhizopus (panel j), Mucor (panel k) and cerebral aspergillosis (panel l) at different magnification levels (Sidebars: 500 µm, and 100µm). At least four mice were used for each group for all experiments. Lesions are highlighted with arrows and outlined in yellow. Data are expressed as median and interquartile range (IQR). Statistical analyses were performed using a two-tailed Mann-Whitney U test. Abbreviations: SUV mean: Standardized uptake value mean; %ID/g: Percentage of Injected Dose per gram of tissue. LPS: Lipopolysaccharide.

**Conclusion:**

^18^F-FDS PET is a promising, noninvasive diagnostic tool for detection and localization of invasive mold infections in several sites. Notably, Gram-negative, Enterobacterales bacteria also metabolize sorbitol, however, patient characteristics, and response to empiric antibiotics can help distinguish bacterial versus mold infections. ^18^F-FDS PET represents an innovative approach to a growing diagnostic challenge in vulnerable patients, with potential to improve diagnosis and treatment, monitor responses, and enable individualized care.

**Figure 4.**
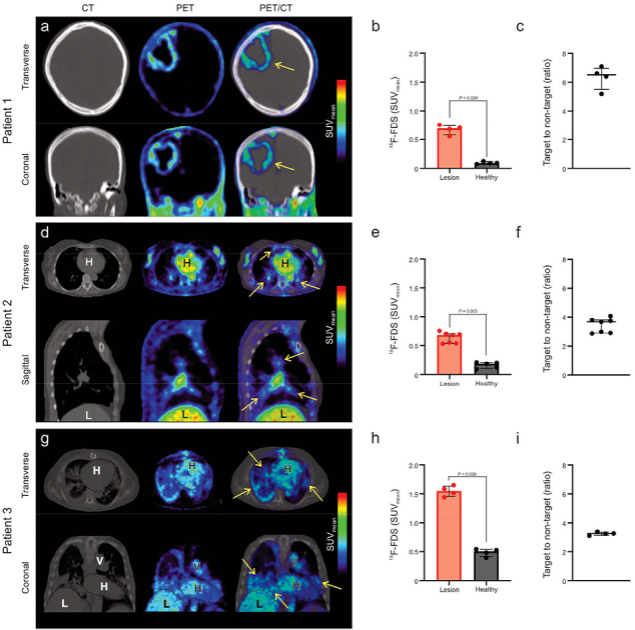
First-in-human 18F-FDS PET in patients with invasive aspergillosis.

18F-FDS PET was performed in three patients with invasive aspergillosis in accordance with U.S. FDA guidelines recruited from an ongoing study approved by the Johns Hopkins Hospitals IRB (clincialtrials.gov NCT05611892). The PET signal is clearly visualized (and quantifiable) in a 35-year-old male with refractory Hodgkin lymphoma and cerebral aspergillosis (panel a-c), a 67-year-old female with nodular pulmonary aspergillosis (panels d-f) and in a 55-year-old male with long term corticosteroid and immunosuppressive treatment with invasive pulmonary aspergillosis due to an azole-resistant Aspergillus calidoustus (panels g-i). 18F-FDS accumulates substantially at the infection sites with a target to non-target ratio of > 3 in all patients. Lesions are pointed with yellow arrows. Data are expressed as median and interquartile range (IQR). Statistical analyses were performed using a two-tailed Mann-Whitney U test. Abbreviations: SUV mean: Standardized uptake value mean. H: heart. L: liver. V: vessels.

**Disclosures:**

Sanjay K. Jain, MD, Fujirebio Diagnostics, Inc. (Malvern PA): Grant/Research Support|Novobiotics, LLC: Advisor/Consultant|Novobiotics, LLC: Grant/Research Support|T3 Pharmaceuticals (Basel, Switzerland): Grant/Research Support

